# A Multilayered GaAs IPD Resonator with Five Airbridges for Sensor System Application

**DOI:** 10.3390/mi15030367

**Published:** 2024-03-08

**Authors:** Xiao-Yu Zhang, Zhi-Ji Wang, Jian Chen, Eun-Seong Kim, Nam-Young Kim, Jong-Chul Lee

**Affiliations:** 1 Department of Electronic Convergence Engineering, Kwangwoon University, Seoul 139-701, Republic of Korea; xyzhang623@foxmail.com; 2 Radio Frequency Integrated Circuit Center, Kwangwoon University, Seoul 139-701, Republic of Korea; zhiji-wang@hotmail.com (Z.-J.W.); cnjacobiii@hotmail.com (J.C.)

**Keywords:** microwave resonator, integrated passive device, gallium arsenide, airbridges

## Abstract

This work proposes a microwave resonator built from gallium arsenide using integrated passive device (IPD) technology. It consists of a three-layered interlaced spiral structure with airbridges and inner interdigital structures. For integrated systems, IPD technology demonstrated outstanding performance, robustness, and a tiny size at a low cost. The airbridges were made more compact, with overall dimensions of 1590 × 800 µm^2^ (0.038 × 0.019 λg^2^). The designed microwave resonator operated at 1.99 GHz with a return loss of 39 dB, an insertion loss of 0.07 dB, and a quality factor of 1.15. Additionally, an experiment was conducted on the properties of the airbridge and how they affected resistance, inductance, and S-parameters in the construction of the resonator. To investigate the impact of airbridges on the structure, E- and H-field distributions of the resonator were simulated. Furthermore, its use in sensing applications was explored. Various concentrations of glucose solutions were used in the experiment. The proposed device featured a minimum detectable concentration of 0.2 mg/mL; high sensitivity, namely, 14.58 MHz/mg·mL^−1^, with a linear response; and a short response time. Thus, this work proposes a structure that exhibits potential in integrated systems and real-time sensing systems with high sensitivity.

## 1. Introduction

In radiofrequency (RF) and microwave systems, the resonator is a crucial component that guarantees excellent operation performance. An RF/microwave resonator typically consists of a two-port network that facilitates frequency selection. In a conventional RF/microwave resonator, components such as coaxial lines, microstrip lines, and rectangular/circular waveguides are frequently utilized. Microwave resonators have been widely used in numerous functional circuit systems throughout the last few decades [1,2,3,4,5]. However, the majority of these resonators require a large area.

It has been difficult for conventional technologies to meet the growing need of miniaturization for RF/microwave systems. Consequently, a number of manufacturing techniques for miniaturization have been thoroughly studied in the past few years, including the use of monolithic microwave integrated circuits [6,7,8,9], substrate-integrated waveguides [10,11,12,13], low-temperature co-fired ceramics [14,15,16,17], and high-temperature superconductors [18,19,20,21]. However, to obtain a low-cost high-performance integrated system, the industry requires consistently compact, reliable, and stable technologies. The integrated passive device (IPD) process is a recent manufacturing technology that has been proposed as a solution for use in RF/microwave device fabrication. It provides simplified and compact modules with high performance through the integration of components with small parasitic effects [22,23,24,25,26,27,28,29,30]. 

Due to their great sensitivity, fast response times, resilience, reusability, and cost-effectiveness, microwave resonators are widely used in sensing systems as well as RF/microwave communication systems [31,32,33,34,35,36,37,38,39,40,41,42,43,44,45,46,47,48,49,50,51]. Common microwave technologies used in sensing systems include parallel plate capacitors, microstrip lines, and cavity resonators [30,31,32,33,34,35,36,37,38,39,42,45,46]. The basic mechanism for which microwave resonators are applied in sensing systems is in detecting the dielectric constant of an object. As a result, a number of sensors operating via dielectric characterization have been proposed, and employing microwave resonators to detect the material under test (MUT) has drawn a lot of attention in recent years [30,34,35,36,37,38,39,40,41,42,43,44,47,48,49,50]. For accurate measurements, microwave resonators use highly sensitive structures, including split-ring resonators (SRRs), interdigital structures, spiral resonators, and airbridges. SRRs exhibit a highly intense electric field at resonance, which is highly sensitive to the sample loading or geometry alteration [39,40]. The primary basis for the interdigital structure employed in sensing is the impact of the material being tested on the capacitance that occurs across the gap in thin-film conductors [36,42]. The coupling capacitor is easily affected by the MUT in a spiral sensor [43,44]. The environment around an airbridge will have a significant impact on its parallel plate capacitance [30,36,37,38]. Frequency, quality factor (Q), phase, and insertion/return loss are frequently utilized parameters from the sensing response and are eventually associated with the MUT. Sensors based on using frequency as the sensing parameter related to the MUT rely on the relationship between resonant frequency and effective permittivity [34,36,37,38,39,40,41,42]. Q is affected by both the effective permittivity and dielectric loss of the MUT and is related to the −3 dB bandwidth [48,49,50]. Similarly, the phase velocity depends on the effectivity permittivity: a change in the effective permittivity results in a change in the phase of the reflection coefficient [35,36]. The sensors monitoring changes in insertion/return losses are primarily governed by changes in the dielectric losses in the MUT induced by fluctuations in glucose concentrations [37,38,50]. Sensing applications can be realized using individual parameters or a combination of them. As previously stated, the increasing demand for RF/microwave systems has outpaced the capabilities of conventional technologies. Contemporary integrated sensing systems need sensors with features like smallness, reliability, stability, and faster response times in addition to high sensitivity, resilience, reusability, and cost-effectiveness. As a result, sensors built from microwave resonators need to be updated for the rapidly developing integrated systems. 

A three-layer microwave resonator based on gallium arsenide (GaAs) with a circular interlaced spiral structure (CSISS), five airbridges, and inner interdigital structures is proposed herein. The proposed resonator was designed for application in modern integrated systems since the three-layered CSISS with airbridges is smaller while also capable of maintaining good performance. The proposed microwave resonator sensor is thoroughly elaborated in this paper. The significance of the airbridges in terms of both miniaturization and sensing is demonstrated comprehensively. Firstly, the CSISS and airbridges are introduced, and the influence of the airbridges on resonator operation was demonstrated considering structure inductance, resistance, and *S*-parameters. In addition, the relationship between *S*-parameters and the dimensions of the resonator are investigated. We fabricated the resonator on a GaAs substrate, which is frequently used in semiconductors for RF/microwave systems [52] because of its semi-insulative properties, low conductor loss, and high Q. The IPD fabrication of the resonator was comprehensively demonstrated for packaging in a quad flat no-lead box with dimensions of 4 × 4 mm^2^. Further, the distribution of E and H fields in the resonator was simulated to explore the effect of airbridges on the structure, indicating the resonator’s susceptibility to permittivity changes. Moreover, to further investigate the possibility of using the resonator in sensing systems, tests using glucose solutions (0.2–5 mg/mL) capable of inducing both hyperglycemia (a glucose concentration > 1.2 mg/mL) and hypoglycemia (a glucose concentration < 0.8 mg/mL) were conducted. The positive experimental results confirmed the feasibility of using the proposed sensor in real-time glucose-concentration-monitoring systems owing to its high sensitivity, real-time response (<1 s), robustness, reliability, reusability, and low cost.

## 2. Design and Analysis

### 2.1. Design of the Proposed Microwave Resonator

**Figure 1:**
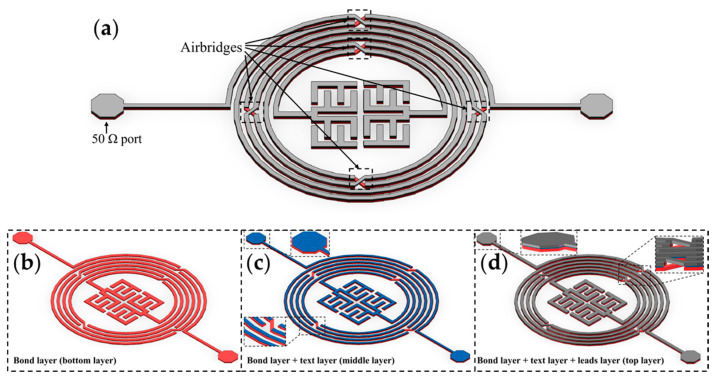
The structure of the proposed resonator with enlarged views. (**a**) Three-dimensional layout of the resonator. (**b**) The bond layer (bottom layer) of the resonator. (**c**) The bond layer and text layer (middle layer) of the resonator. (**d**) The bond and text layers and lead layer (top layer) of the proposed resonator.

**Figure 2:**
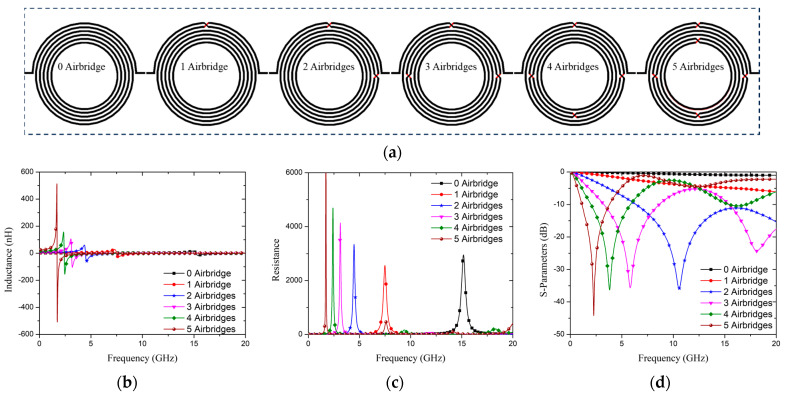
(**a**) Diagram of three-layer CSISS with 0–5 airbridges. Parameters of three-layer CSISS with 0–5 airbridges: (**b**) inductance, (**c**) resistance, and (**d**) S-parameters.

Owing to their high inductance, low loss, and simplicity in terms of integration, circular or rectangular spiral inductors are frequently utilized in integrated RF/microwave systems. A rectangular spiral inductor is easier to design, whereas a circular one exhibits a higher self-resonant frequency [52]. However, with advancements in technology, conventional techniques have failed to satisfy the growing demands of miniaturization. Therefore, this study proposes a microwave resonator comprising three laminated conductor layers with five airbridges and two series-wound interdigital capacitors in the middle of a CSISS, as shown in Figure 1a. It comprises bond, text, and lead layers from bottom to top. Figure 1b shows the bottom layer (bond layer) of the proposed resonator; the bond layer was covered by the middle layer (text layer), as shown in Figure 1c. As shown in Figure 1d, the top layer (leads layer) was used to cover the text layer. Traditionally, airbridges have been used to connect two non-connecting transmission lines and reduce the shunt capacitance between conductors and the ground plane in multilayer spiral inductors [24,30,53]. The five airbridges proposed in this study linked all the conductors and reduced parasitic capacitance (an unavoidable and usually unwanted capacitance). The parallel plate capacitance of an airbridge is expressed as [52]

(1) $$C=\frac{\epsilon_{0}\epsilon_{d}A}{d},$$

where *ϵ_d_* is the dielectric constant between plates, *A* is the overlap area, and *d* is the distance between two conductors. Thus, it is possible to insert materials with different dielectric constants between the plates to adjust the parallel plate capacitance. A schematic of the three-layer CSISS with 0–5 airbridges is shown in Figure 2a, and the S-parameter, resistance, and inductance of the structures with 0–5 airbridges are shown in Figure 2b–d, respectively. The resonant frequencies of the structures with two, three, four, and five airbridges were 10.56, 5.82, 3.81, and 2.28 GHz, respectively, as shown in Figure 2d, whereas the structures with zero and one airbridges did not resonate at 0–20 GHz. More airbridges resulted in improved S-parameters. The S_11_ (return loss) values in the stopband for two, three, four, and five airbridges structures were −11.08, −5.24, −2.54, and −1.07 dB, and the resonant frequency decreased. Based on the same fabrication settings, resonant frequency has a reciprocal relationship with chip size [54]. Therefore, for a fixed frequency, the airbridge will induce a size reduction with a performance improvement. Considering the *Y*-parameter, the equivalent resistance and inductance of the structure are respectively calculated as follows:

(2) $$R\left(\Omega \right)=real\left(\frac{1}{Y(1,1)}\right),$$

(3) $$L\left(nH\right)=\frac{1.0\times 10^{9}\times imag\left(\frac{1}{Y\left(1,1\right)}\right)}{2\pi f},$$

where *Y* is admittance, *f* is the operating frequency, and *imag* and *real* represent the imaginary and real parts, respectively. The Supplementary Materials (Figure S1) contain further information regarding this methodology, including the rationale behind the use of a three-layer structure.

While the multilayered CSISS with five airbridges generated inductance and capacitance to achieve resonance, the inner interdigital structure compensated for the capacitance and inductance. Figure S2 displays the simulated capacitance and S-parameters of the structure with and without the inner interdigital structure. S-parameter performance was enhanced and the resonant frequency was adjusted through the usage of an inner interdigital structure. Refer to Figure S2 for more details.

### 2.2. Simulation and Optimization

**Figure 3:**
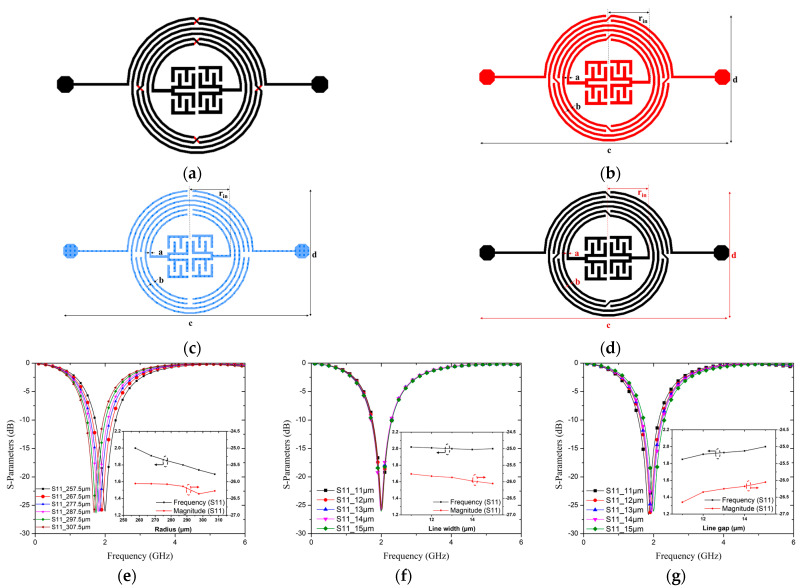
(**a**) Schematic of proposed resonator and its (**b**) bond, (**c**) text, and (**d**) lead layers. Simulated S_11_ according to frequency for varying (**e**) inner radius *r*_in_, (**f**) line width *a*, and (**g**) line gap *b*.

The topologies of the three layers are shown in Figure 3a–d. The effects of the resonator’s geometric parameters, including its inner radius *r*_in_, width *a*, and gap *b*, were explored experimentally. The inner radius *r*_in_ varied from 257.5 to 307.5 μm with increments of 10 μm. The width *a* of the CSISS for the bond and lead layers varied from 15 to 11 μm at intervals of 1 μm, and that for the text layer ranged from 11 to 6 μm. Finally, Gap *b* between the CSISS transmission lines of the bond and lead layers varied from 15 to 11 μm in intervals of 1 μm, whereas that of the text layer decreased from 19 to 15 μm. Figure 3e–g illustrate the parameter S_11_ based on the resonant frequency for a varying inner radius *r*_in_, width *a*, and gap *b* in the CSISS. With an increase in *r*_in_ from 257.5 to 307.5 μm, the frequency of the pole changed from 2.0 to 1.69 GHz, and S_11_ changed from −26.05 to −26.27 dB, as shown in Figure 3e. In Figure 3f, the frequency of the pole varied from 2.02 to 2.0 GHz, and S_11_ ranged from −25.76 to −26.05 dB with an increase in the line width from 11 to 15 μm. As shown in Figure 3g, with an increase in the line gap from 11 to 15 μm, the pole frequency increased from 1.85 to 2.0 GHz, and S_11_ increased from −26.65 to −26.05 dB. We can see that the radius has a big influence on resonant frequency and affects the magnitude of S_11_ slightly. The line gap affects both the resonant frequency and magnitude of S_11_ a lot. Line width has a minor effect on the magnitude of S_11_, while it barely affects the resonant frequency. It is very challenging to determine the equivalent inductance, capacitance, and precise geometric characteristics for a given frequency for a complex three-layered spiral structure with five airbridges. As a result, we employed the ADS to resolve this issue. We repeatedly tuned the resonator’s dimensions within the limitations of the fabrication process (pertaining to the thickness of each layer, line width, and gap), balancing the resonator’s performance with the minimization of size and obtaining the ideal geometric characteristics. Table 1 lists the geometric parameters of the resonator. The resonant frequency was set as 2 GHz, a value constituting a tradeoff between measurement and fabrication.

## 3. Fabrication and Measurement

### 3.1. IPD Fabrication 

**Figure 4:**
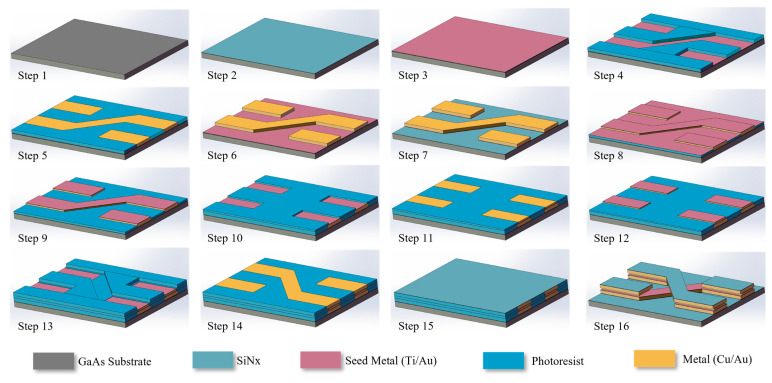
Diagram of GaAs-based IPD nanofabrication.

Figure 4 illustrates the GaAs-based IPD nanofabrication of the proposed resonator. In step 1 of wafer cleaning, ionic pollutants, organic impurities, and native chemical oxides were treated and removed from the GaAs substrate using an ultrasonic acetone bath, isopropyl alcohol, and deionized water. In step 2 of wafer passivation (SiNx), a 0.2 μm SiNx layer (relative permittivity, 7.5; loss tangent, 0.002) was deposited as a passivation layer to create an even wafer surface free of roughness and other imperfections using plasma-enhanced chemical vapor deposition. In step 3 of seed metal (Ti/Au) sputtering, deposition was conducted in a chamber with a 1:19 mixture of SiH_4_ and NH_3_ at a temperature of 250 °C, a chamber pressure of 1200 mTorr, a 2000 gas flow, and 100 W of RF power. To facilitate strong adhesion between the substrate and first metal layer, 20 and 80 nm thick Ti and Au seed layers, respectively, were built using the sputtering procedure following passivation layer deposition. In step 4, the first metal layer with a photoresist was created through the spinning of the photoresist onto the wafer using a spin coater, followed by an exposure and development procedure applied to define the first metal layer. In step 5, the first layer (i.e., the bond layer) was fabricated by plating 4.5 and 0.5 μm thick Cu and Au layers, respectively. The electron energy was fixed at 10 kV, and deposition was conducted at a pressure of 5.010–6 mTorr. In addition, a minimum deposition rate of 0.5 Å/s was set to achieve layer thickness precision. Further, in step 6 of photoresist removal, acetone, isopropyl alcohol, and deionized water were used in a lift-off machine for 90 s. Step 7 involved dry etching of the seed metal after lift-off, wherein the undesired seed metal area was removed using inductively coupled plasma dry etching (SF6/Ar). Step 8 was seed metal (Ti/Au) sputtering, wherein the photoresist was coated onto the wafer again, following which the second seed metal layer (20/80 nm Ti/Au layers) was constructed. Step 9 involved a seed metal dry-etching procedure similar to that conducted in step 7. Thereafter, step 10 was the fabrication of a second metal layer photoresist, wherein photoresist coating, exposure, and development were performed in sequence for airbridge post-patterning. In step 11 of the metal plating of the second layer (i.e., the text layer), we used hard baking to reflow the airbridge post-pattern at a temperature of 130 °C for 180 s. Subsequently, the second metal layer was fabricated (with 1.6 and 0.2 µm thick Cu and Au layers, respectively) via electroplating. Step 12 involved seed metal (Ti/Au) sputtering carried out in a manner similar to that in step 3. Then, Step 13 was the fabrication of the third metal layer photoresist, whereas step 14 was third-layer (i.e., the lead layer) metal plating. In step 15 of wafer passivation (SiNx), a 0.2 µm thick SiNx layer was deposited. Finally, in step 16, the photoresist was removed with acetone, isopropyl alcohol, and deionized water using a lift-off machine. High-quality GaAs substrates, excellent processing accuracy, and muti-layer fabrication capabilities facilitate IPD technology’s widespread use in RF/microwave integrated systems for balancing tradeoffs and providing simplified and compact modules with high performance via the integration of components with small parasitic effects. 

### 3.2. Measurements of the Proposed Microwave Resonator

**Figure 5:**
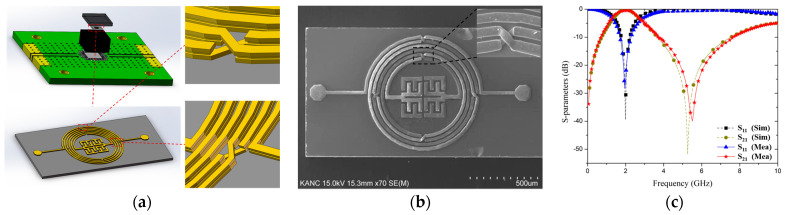
(**a**) Three-dimensional layout of the resonator (three laminated layers) with partially enlarged views. (**b**) SEM image of fabricated resonator. (**c**) Simulation results and measurements (S-parameters) for the proposed resonator.

The resonator was simulated with the Advanced Design System (ADS) and built on a GaAs substrate with a dielectric constant, thickness, and tanδ of 12.85, 200.1 μm, and 0.006, respectively. The resulting resonator dimensions were 1590 × 800 μm^2^ (0.038 × 0.019 λg^2^). Figure 5a shows the three-dimensional layout of the proposed quad flat no-lead-packaged resonator with partially enlarged views. A scanning electron microscopy (SEM) image of the top of the fabricated resonator is shown in Figure 5b. A vector network analyzer (VNA) was used to measure the S-parameters of the resonator. The chip was wire-bonded on a printed circuit board, and the two ports were connected to the analyzer via subminiature version A connectors.

The simulation results and measurements of the S-parameters of the proposed IPD resonator are shown in Figure 5c. In the simulation, the operating frequency in the passband was 2.0 GHz, a value representing a tradeoff between measurement and fabrication, and the insertion and return losses were 0.071 and 39 dB, respectively. Further, in the measurements, the center frequency was 1.99 GHz, and the insertion and return losses were 0.45 and 28.33 dB, respectively. The −3 dB passband of the fabricated resonator ranged from 1.25 to 2.98 GHz, with a fractional bandwidth of 86.93% and a Q of 1.15. There are minor differences between the simulated and measured results, mainly at the point of zero transmission of S_21_, which might be due to the fabrication and measurement process errors, but they are acceptable. Table 2 presents a comparison of the proposed GaAs-based IPD resonator with resonators based on other technologies [6,10,15,21,22] and a GaAs-based IPD resonator without airbridges [28]. The table exhibits the merits of a wide band, a reduction in size, and good performance regarding insertion and return loss of the proposed resonator, demonstrating its potential for applications in integrated microwave systems.

## 4. The Proposed Microwave Resonator in Sensing Applications

### 4.1. Electromagnetic Field Distribution of the Proposed Microwave Resonator

**Figure 6:**
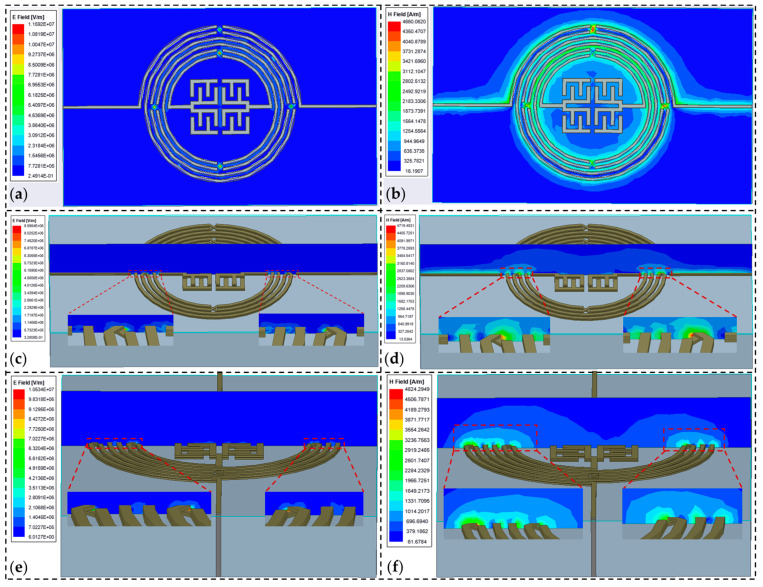
(**a**) E- and (**b**) H-field distributions in the plane parallel to the resonator. (**c**,**e**) E- and (**d**,**f**) H-field distributions and their enlarged views in planes perpendicular to the resonator.

The High-Frequency Structure Simulator (HFSS) was used to simulate the E- and H-field distributions to further investigate the effect of airbridges on the proposed microwave resonator. Figure 6a,b show the E- and H-field distributions on a plane parallel to the resonator, respectively, corresponding to the location of the airbridge. The E- and H-field distributions for planes perpendicular to the resonator plane, along with magnified views of them for planes crossing the centerline of the airbridge, are displayed in Figure 6c–f. The E- and H-fields were concentrated on the airbridge, indicating that this structure is more sensitive to changes in environment, i.e., dielectric constant fluctuations. Equation (1) states that every parameter for a fabricated resonator, with the exception of the dielectric constant between the plates (*ϵ_d_*), has a fixed value. Therefore, the capacitance value will change according to how the value of *ϵ_d_* varies with different environmental conditions. According to Equation [55] below

(4) $$f_{0}=\frac{1}{2\pi \sqrt{LC}},$$

the resonator with airbridges could be used as a frequency-based sensor [30], which will be evaluated in the following experiment.

### 4.2. Experiments Regarding the Proposed Microwave Resonator’s Capacity for Glucose Detection

**Figure 7:**
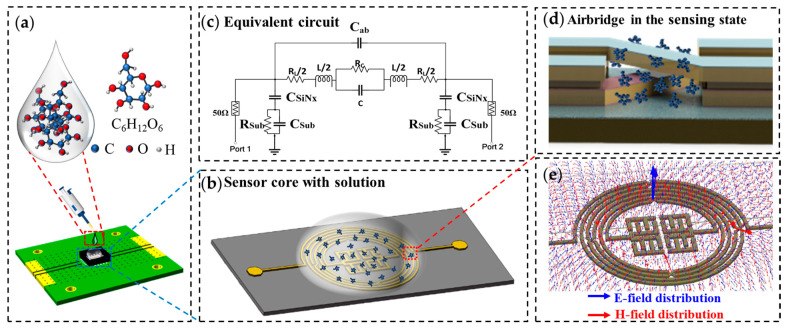
The glucose detection mechanism of the proposed resonator: (**a**) 3D schematic of the experiment, (**b**) sensor core with glucose solution, (**c**) schematic of the proposed IPD resonator, (**d**) an enlarged view of the airbridge in the sensing state, and (**e**) E- and H-field distributions of the resonator.

**Figure 8:**
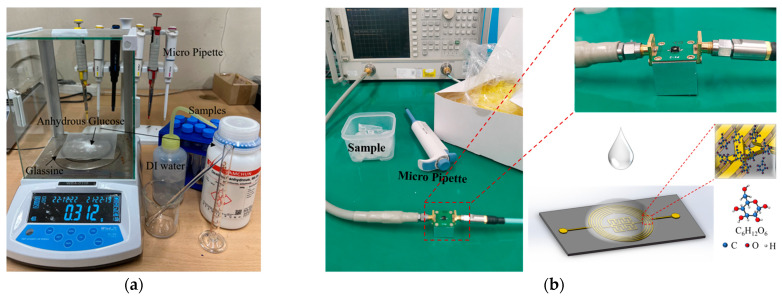

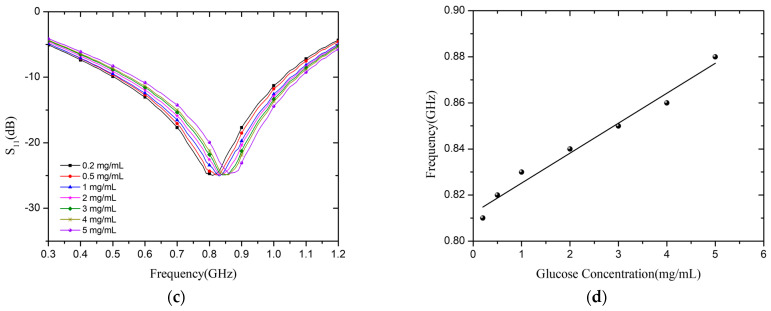
(**a**) Preparation of the glucose solution. (**b**) The glucose-sensing mechanism of the proposed sensor. (**c**) S-parameters (S_11_) with various concentrations of glucose solutions. (**d**) The center frequency of S_11_ with various glucose concentrations.

To investigate the sensing capabilities of the designed microwave resonator, glucose-based solutions [56] were prepared by quantizing and combining anhydrous glucose with deionized (DI) water at different concentrations ranging from 0.2 to 5 mg/mL (0.2, 0.5, 1, 2, 3, 4, and 5 mg/mL) to incorporate cases of diabetes patients with hyperglycemia (a glucose concentration > 1.2 mg/mL) and hypoglycemia (a glucose concentration < 0.8 mg/mL). The glucose detection mechanism of the proposed resonator is shown in Figure 7. The process of preparing glucose solutions is introduced in Figure 8a; refer to the Supplementary Materials for more details on the solution preparation. The solutions were prepared and then utilized immediately for the experiment. Figure 8b depicts the glucose-sensing mechanism of the microwave resonator sensor. The S-parameters of the proposed sensor with the glucose solution were measured using a VNA. As demonstrated by the E- and H-field distributions in Figure 6, the proposed resonator, particularly the airbridge region, was sensitive to permittivity fluctuations. The samples of the pre-prepared glucose solution were dropped using a micropipette, and each sample was tested at a room temperature of 25 °C in a fixed volume of 5 µL. To guarantee reliable data collection, DI water was added to the sensor before each test to flush out any residual glucose solution. Figure 8c shows the S-parameters (S_11_) of the sensor at different glucose solution concentrations. The graph clearly shows a linear relationship between the resonant frequency and the glucose concentration. The sensor that was demonstrated had a minimum measurable concentration of 0.2 mg/mL. We measured the glucose solution at 0.1 mg/mL: there was no discernible frequency shifting from the DI water; however, the glucose solution at 0.2 mg/mL clearly displayed frequency shifting, as seen in Figure S3. Additionally, the center frequency of S_11_ ranged from 0.81 to 0.88 GHz for a glucose concentration range of 0.2 to 5 mg/mL, with a high sensitivity of 14.58 MHz/mg·mL^−1^. Figure 8d shows that the glucose concentration exhibited a good linear relationship with the center frequency of S_11_, and the response time was less than 1 s. Table 3 presents a comparison of the proposed glucose sensor with sensors from previous works. Based on the IPD microwave resonator, a glucose sensor with high sensitivity, real-time response capacity, durability, dependability, reusability, and low cost was created.

Although this study is limited in that we did not prove that the proposed sensor could be used for glucose detection in a non-invasive way, e.g., by attaching the device to the human body, we did show the sensing capability of this device, and it can be used in more ways in the future by utilizing non-invasive samples like urine, saliva, etc. Additionally, we investigated the potential for this device to function as a non-invasive device using simulations. More improved and lucid E- and H-filed distributions are shown in Figure S4. As a result, future research on microfluidics for non-invasive sensing will be conducted.

## 5. Conclusions

This research proposes a microwave resonator based on GaAs, featuring a three-layered CSISS, five airbridges, and an inner interdigital structure. This resonator was fabricated using IPD technology and packed in a quad flat no-lead box. The use of multilayered IPD manufacturing technology led to airbridges enhancing compactness, achieving total dimensions of 1590 × 800 μm^2^ (0.038 × 0.019 λg^2^). Additionally, the measured resonance frequency was 1.99 GHz, accompanied by a fractional bandwidth of 86.93% at −3 dB. Simulations of inductance, resistance, and S-parameters using the ADS showcased how airbridges impact the design of resonators. HFSS facilitated the simulation of E- and H-field distributions, revealing airbridges’ high sensitivity to permittivity variations, thereby showcasing their sensing capabilities. To investigate the resonator’s sensing abilities, glucose solutions ranging from 0.2 to 5 mg/mL were employed, encompassing concentrations for diabetes patients with both hyperglycemia and hypoglycemia. The experiment revealed the resonator had a minimum detectable concentration of 0.2 mg/mL, a high sensitivity of 14.58 MHz/mg·mL^−1^ with a linear response, and a short response time (<1 s). Consequently, the airbridges performed well in integrated systems and contributed to glucose detection. Upcoming research ought to concentrate on improving the selectivity of this sensor as well as the performance of its structure, particularly for airbridges, and broadening its applications in integrated system sensing.

## Figures and Tables

**Table 1:** Geometric parameters of resonator layers.

Parameter	*a*	*b*	*c*	*d*	*r*_in_	Thickness
Bond layer (μm)	15	15	1590	870	257.5	5
Text layer (μm)	11	19	1586	796	257.5	1.8
Leads layer (μm)	15	15	1590	800	257.5	5

**Table 2:** Comparison of proposed microwave resonator with technologies from other works.

Ref.	Technology	*f*_0_ (GHz)	Fractional Bandwidth (%)	Insertion Loss (dB)	Return Loss (dB)	Size
[6]	Monolithic microwave integrated circuit	28.6	17.5	0.95	–	$0.00158\;\lambda_{g}^{2}$
[10]	Substrate-integrated waveguide	3.5	16	0.91	20	$1.2\;\times \;0.8\;\lambda_{g}^{2}$
[15]	Low-temperature co-fired ceramics	2.4	12.5	–	>15	$0.058\;\times \;0.058\;\times \;0.011\;\lambda_{g}^{3}$
[21]	High-temperature superconductor	0.143	0.99	0.43	–	$0.033\;\times \;0.017\;\lambda_{g}^{2}$
[22]	Glass IPD	2.6	49.62	0.6	>30	$0.018\;\times \;0.009\;\lambda_{g}^{2}$
[28]	GaAs IPD (Without airbridges)	11.2	14.3	1.3	20.9	3860 × 2500 μm^2^$(0.51\;\times \;0.33\;\lambda_{g}^{2}$)
This work	GaAs IPD	1.99	86.93	0.45	28.33	$0.038\;\times \;0.019\;\lambda_{g}^{2}$

**Table 3:** Comparison of proposed glucose sensor with sensors from other works.

Ref.	Technology	*f*_0_ (GHz)	Sensing Parameter	Concentration Range	Minimum Detection	Sensitivity
[36]	IPD Spiral structure Airbridge	3.18	f, PH	50–250 mg/dL	50 mg/dL	0.32 MHz/mg/dL
[39]	SRR	–	f, RL	0–5 mg/mL	1 mg/mL	0.50 MHz/mg/mL 0.50 dB/mg/mL
[40]	SRR	2.395	f	67–400 mg/mL	67 mg/mL	0.72 MHz/mg/mL
[41]	SRR, interdigital	4.18	f	0–5000 mg/mL	1250 mg/mL	2.60 × 10^−2^ MHz/mg/mL
[42]	CPW, Interdigital	–	f, IL	0–1 g/mL	0.11 g/mL	235.32 MHz/g/mL 15.30 dB/g/mL
[44]	Spiral structure	7.65	f, RL	50–600 mg/mL	50 mg/mL	−0.022 dB/mg/dL
[56]	IPD Spiral structure Airbridge	0.8, 3.2	f	25–300 mg/dL	25 mg/dL	1.38 MHz/mg/dL
This work	IPD Spiral structure Airbridge	1.99	f	0.2–5 mg/mL	0.2 mg/mL	14.58 MHz/mg/mL

PH: phase. RL: return loss. IL: insertion loss. CPW: coplanar waveguide.

## Data Availability

Data are contained within the article.

## Supplementary Materials

The following supporting information can be downloaded at https://www.mdpi.com/article/10.3390/mi15030367/s1. Figure S1: (a) Geometry and (b) inductances of the one-layer, three-layer, and three-layer with five airbridges structures. Figure S2: (a) Capacitance and (b) S-parameters of the resonator with and without an inner interdigital structure. Figure S3: The center frequency of S_11_ with DI water and 0.1 and 0.2 mg/mL glucose solutions. Figure S4: E- and H-field distributions in planes perpendicular to the resonator.
