# Supplementary material for: A Multilayered GaAs IPD Resonator with Five Airbridges for Sensor System Application
Source: Micromachines (Basel). 2024 Mar 8;15(3):367. doi: 10.3390/mi15030367 (PMC10972498; doi:10.3390/mi15030367)
Supplement: Supplementary file 1 [file micromachines-15-00367-s001.zip › micromachines-2872985-supplementary.pdf]

micromachines

MDPI

Supplementary Materials

A Multilayered GaAs IPD Resonator with Five Airbridges for Sensor System Application

Xiao-Yu Zhang [1], Zhi-Ji Wang [2], Jian Chen [2], Eun-Seong Kim [2,*], Nam-Young Kim [2,*] and Jong-Chul Lee [1,*]

[1] Department of Electronic Convergence Engineering, Kwangwoon University, Seoul 139-701, Republic of Korea; xyzhang623@foxmail.com
[2] Radio Frequency Integrated Circuit Center, Kwangwoon University, Seoul 139-701, Republic of Korea; zhiji-wang@hotmail.com (Z.-J.W.); cnjacobiii@hotmail.com (J.C.)
* Correspondence: esk@kw.ac.kr (E.-S.K.); nykim@kw.ac.kr (N.-Y.K.); jclee@kw.ac.kr (J.-C.L.)

The equation of resonant frequency is $f_0 = 1/2\pi\sqrt{LC}$, where f_0 is the resonant frequency, L and C denote the inductance and capacitance, respectively.

For the fixed chip area, we found that a 3-layer features a higher inductance. The difference of inductance between one-layer, three-layer, and three-layer with five airbridges (the proposed resonator) based on the simulation results in advance design system were demonstrated in Figure S1. As shown in Figure S1a, the size information of these three structures is all the same: inner radius, conductor width, conductor spacing, and the number of turns.

The simulated inductance of these three structures (one-layer structure, three-layer structure, and three-layer structure with five airbridges) is shown in Figure S1b, where we see the highest inductance is achieved by using three-layer structure with five airbridges, while the one-layer just achieve the lowest inductance.

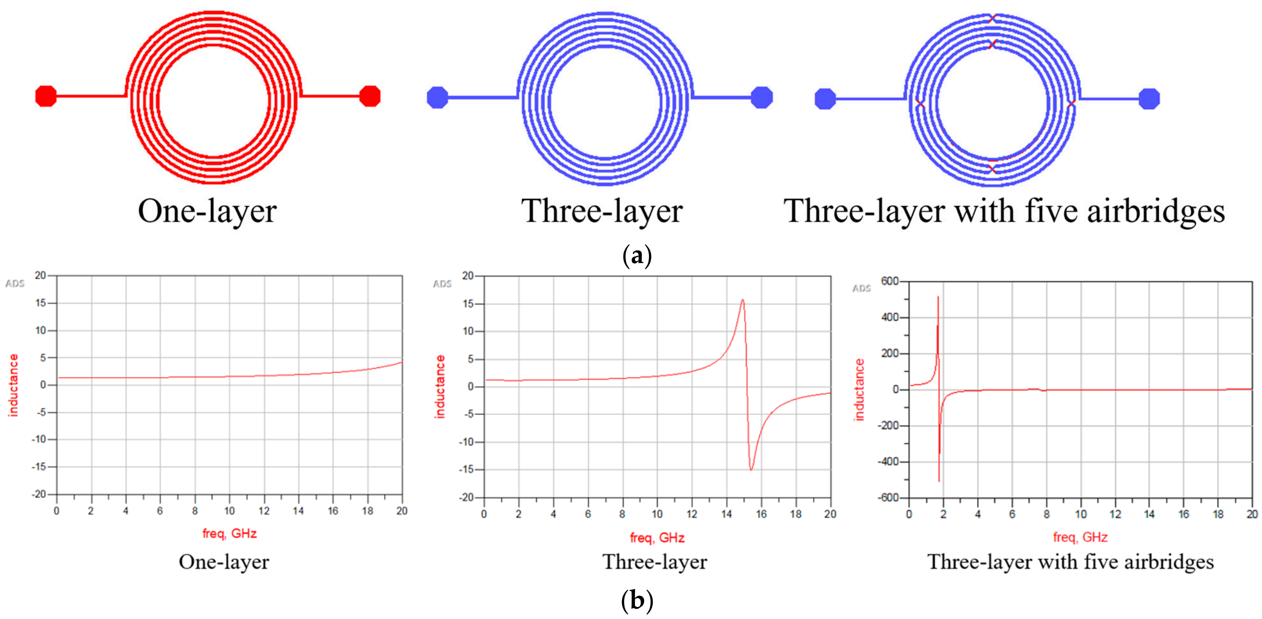

Figure S1. (**a**) Geometry and (**b**) inductances of the one-layer, three-layer, and three-layer with five airbridges structures.

The capacitance and S-parameters of the structure with and without the inner interdigital structure were simulated and shown in Figure S2. From Figure S2a, the interdigital capacitor provides higher capacitance values, which compensate the capacitance for the

proposed resonator according to $f_0 = 1/2\pi\sqrt{LC}$. As a consequence, higher capacitance value decreases the center frequency from 2.28 to 2.0 GHz. In addition, the performance of S-parameters was improved with better suppression out of the working frequency with the transmission zero of S_{21} changed from −10.77 to −46.33 dB, as illustrated in Figure S2b.

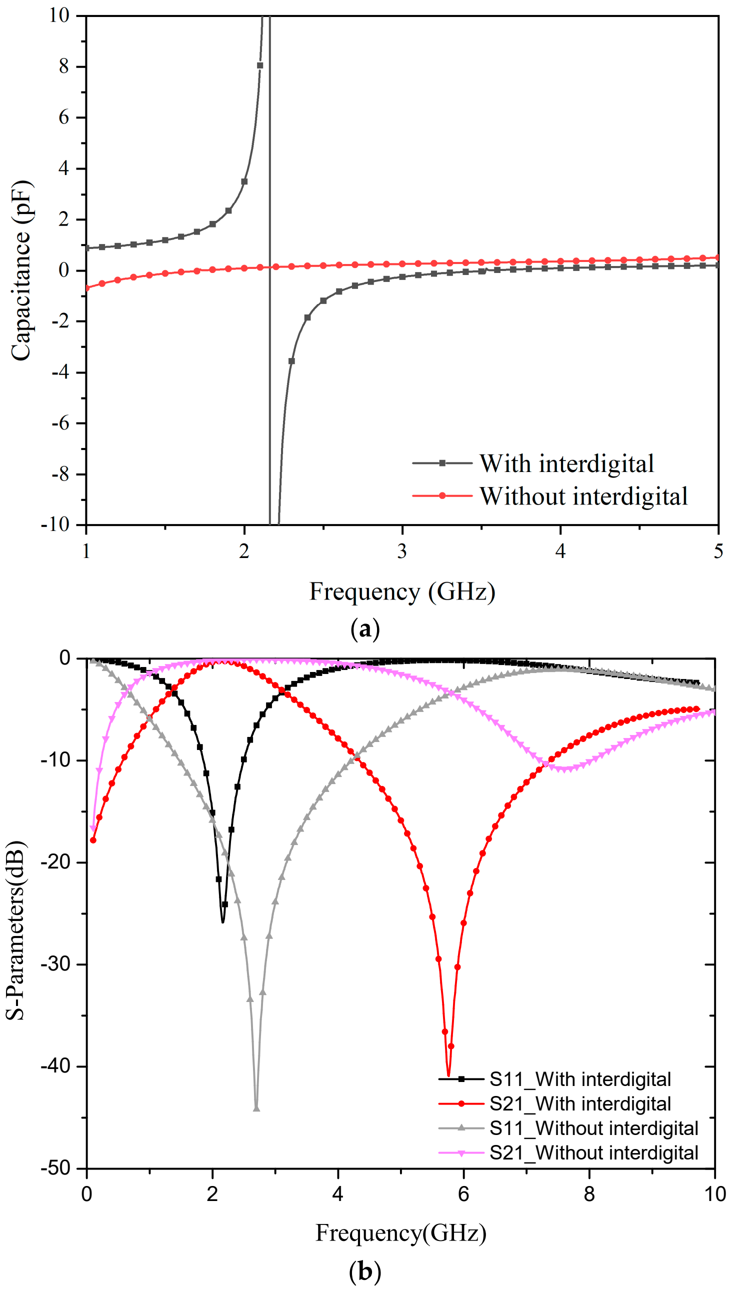

Figure S2. (**a**) Capacitance and (**b**) S-parameters of the resonator with and without the inner interdigital structure.

The initial concentrations of glucose solutions of 0.2, 0.5, 1, 2, 3, 4, 5 mg/mL were prepared as:

- The anhydrous glucose was weighted by a high-precision lab balance (a precision of 0.001 g) with a glassine.
- The deionized (DI) water was quantified by the graduated cylinder and transferred to the beaker.
- Put the anhydrous glucose into the beaker and stir with the glass stirring rod.
- The mixed glucose solutions were stored in tubes.

Errors exist during the process, especially in weighting the anhydrous glucose and quantifying the DI water, however, the high-precision balance and careful operation may control these errors to an acceptable level.

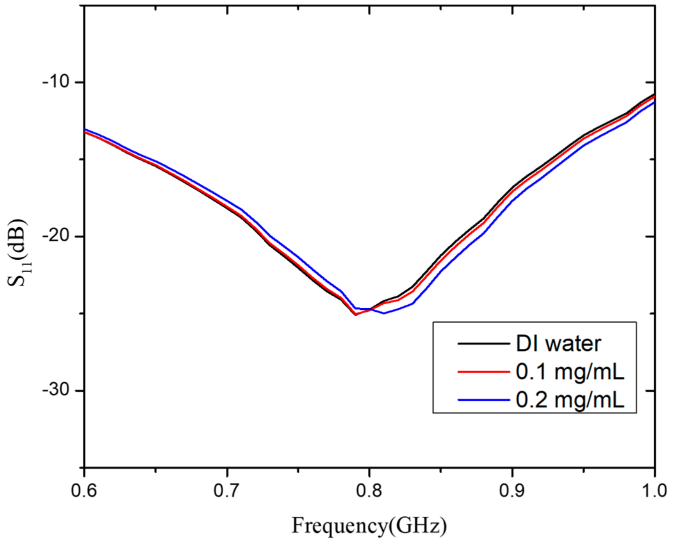

Figure S3. The center frequency of S_{11} with DI water, 0.1, and 0.2 mg/mL glucose solutions. .

We measured the glucose solution with concentration of 0.1 mg/mL, it didn't show a significant frequency shifting from the DI water, while the solution with concentration of 0.2 mg/mL showed an obvious frequency shifting, as shown in Figure S3. Thus, the minimum detectable concentration of the proposed glucose sensor is set as 0.2 mg/mL.

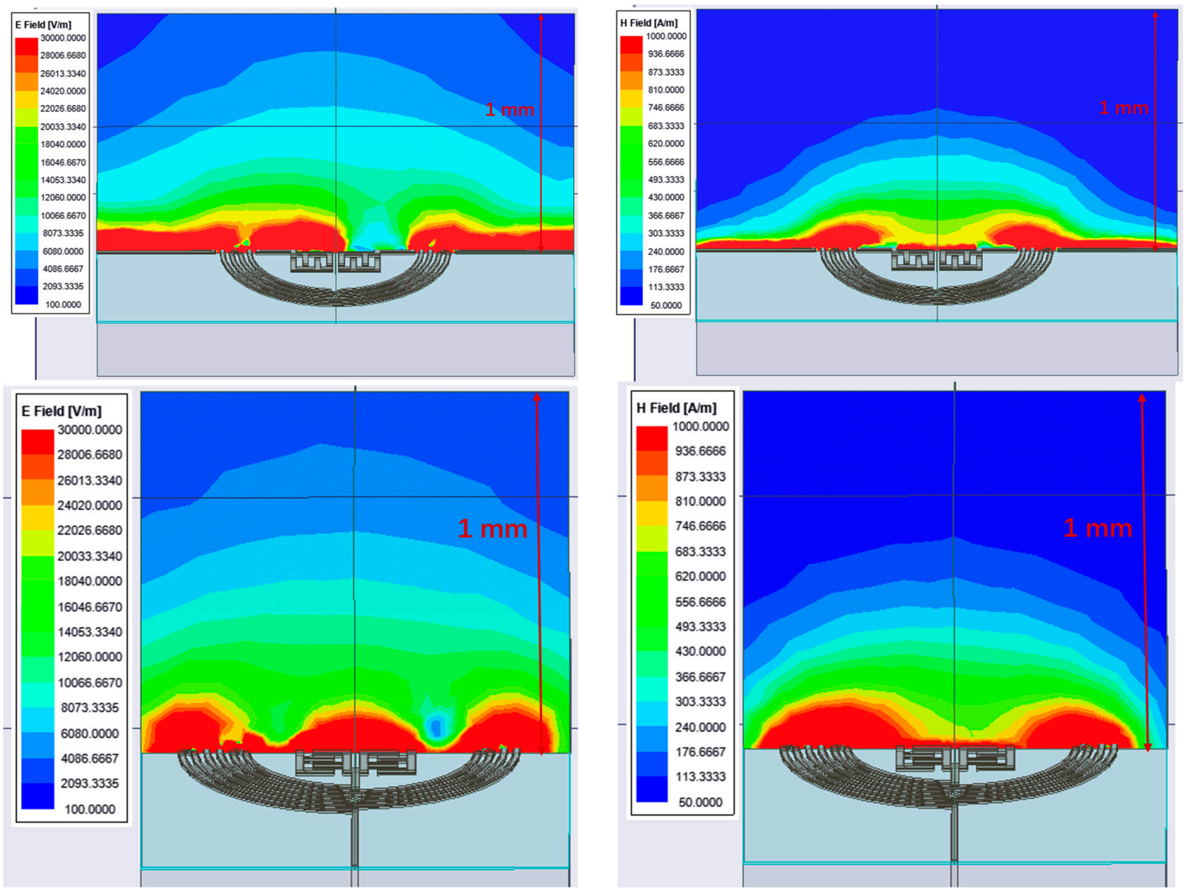

Figure S4. E- and H-field distributions on planes perpendicular to the resonator.

The E- and H-field distributions in the space above the proposed sensor with a distance of 1 mm were simulated and demonstrated in Figure S4. For a better illustration, the scale of the E-field distribution is set as 100–30,000 V/m and that of H- field distribution is 50–1000 A/m. Despite of the strongest electromagnetic fields distributed in the airbridge, there are still sufficient E- and H-field distributions in the space above the proposed resonator especially above the airbridge region.
